# Genomic and Global Approaches to Unravelling How Hypermutable Sequences Influence Bacterial Pathogenesis

**DOI:** 10.3390/pathogens3010164

**Published:** 2014-02-25

**Authors:** Fadil A. Bidmos, Christopher D. Bayliss

**Affiliations:** Department of Genetics, University of Leicester, University Road, Leicester LE1 7RH, UK; E-Mail: fab11@le.ac.uk

**Keywords:** phase variation, simple sequence repeat, phasevariome, phasotype, localised hypermutation, bacterial genome

## Abstract

Rapid adaptation to fluctuations in the host milieu contributes to the host persistence and virulence of bacterial pathogens. Adaptation is frequently mediated by hypermutable sequences in bacterial pathogens. Early bacterial genomic studies identified the multiplicity and virulence-associated functions of these hypermutable sequences. Thus, simple sequence repeat tracts (SSRs) and site-specific recombination were found to control capsular type, lipopolysaccharide structure, pilin diversity and the expression of outer membrane proteins. We review how the population diversity inherent in the SSR-mediated mechanism of localised hypermutation is being unlocked by the investigation of whole genome sequences of disease isolates, analysis of clinical samples and use of model systems. A contrast is presented between the problematical nature of analysing simple sequence repeats in next generation sequencing data and in simpler, pragmatic PCR-based approaches. Specific examples are presented of the potential relevance of this localized hypermutation to meningococcal pathogenesis. This leads us to speculate on the future prospects for unravelling how hypermutable mechanisms may contribute to the transmission, spread and persistence of bacterial pathogens.

## 1. Introduction

Adaptability is a common feature of bacterial pathogens. The rapidly changing milieu of an inflammatory host environment and traversing between host tissues with their differing nutrient contents and innate/adaptive immune effectors requires the induction of a diverse set of defence/attack molecules and metabolic pathways by the bacterial cells. Additionally, strong selective pressures are also exerted on these pathogens by transmission within an immunologically and genetically variable host population. Rapid, stochastic generation of phenotypic variation by “hypermutable” mechanisms is a widespread adaptive phenomenon that has evolved within the genomes of several bacterial pathogens as a response to these forces [1,2,3,4]. The prevalence of these mechanisms in disease isolates and the amounts of variation manifest during disease is amenable to high throughput global analysis. This review will consider the nature of one of these hypermutable mechanisms—simple sequence repeat tracts (SSRs)—examining the experimental techniques utilised for analysis, particularly the utility of next generation sequencing (NGS), and examples of the potential contributions of this mechanism to disease.

## 2. Localised Hypermutation, Phase Variation and the Mechanisms of Hypermutation

The mutability of genomes varies as a function of the DNA sequence and the complement of DNA replication and repair enzymes. The average mutation rate of a bacterial gene is 1 × 10^−9^ mutations/division, but there are certain sequences, such as microsatellites, whose mutation rates are significantly higher than this average [5,6]. Evolution has acted on these sequences, sometimes in combination with trans-acting factors, to evolve loci with mutation rates up to 1 × 10^−3^ per division [7]. This heightened mutability is referred to as localised hypermutation and is contained in ‘contingency’ loci [8]. These loci are thought to have evolved for adaptation to the fluctuating pressures acting on bacterial pathogens.

The major mechanisms of localised hypermutation are site-specific recombination, homologous recombination of tandem duplications of DNA sequences, simple sequence repeats and G-quartet-mediated gene conversion [3,4,8,9,10]. Only one example of this last mechanism has been described, and it mediates antigenic variation in the major pilin sub-unit of *Neisseria* [11,12]. The variation is produced by the insertion of sequences into an expression locus (*i.e.*, the *pilE* gene) from silent partial copies of the gene, producing a vast array of allelic variants. The other mechanisms generate reversible mutations, meaning that there is no loss of genetic information. These hypermutable mechanisms often result in reversible switches in the expression of the associated genes and phenotypes. These stochastic, high frequency, reversible switches in gene expression are responsible for the phenomenon of phase variation (PV), which is widely observed in bacterial pathogens [8,13].

The majority of contingency loci identified to date encode outer membrane proteins (OMPs), enzymes involved in the modification of surface molecules or restriction-modification systems. The known and potential functions of these loci have been extensively reviewed elsewhere [1,2,3,4,10,14]. The main functions of these loci are, however, in mediating interactions between the pathogen and their hosts or altering resistance to phage infection. The contributions of contingency loci to the pathology, course of disease, host persistence, transmission and spread of bacterial pathogens is an expanding area of research relevant to a diverse range of species from *Mycoplasmas*, *Campylobacters*, *Helicobacters*, *Neisseria*, *Bacteroides* and coliforms. This review will consider only SSR-mediated hypermutation and highlight some examples of how research into these types of contingency loci is improving our understanding of aspects of bacterial pathogenesis.

## 3. Detection of Hypermutable Sequences in Genomes

### 3.1. A Definition of Functional SSR Classes

One of the most widespread mechanisms of localised hypermutation involves hypermutable SSR sequences. These sequences are easily detected in genomes; however, there is no consensus on the definition of a ‘functional SSR’ or for classification into different functional classes. We set out below a scheme for separating SSR into different classes based on their potential to frequently and reproducibly mediate switching (*i.e.*, PV). Mutation rate is an important determinant of the ability of an SSR to mediate PV and to facilitate the adaptation of a bacteria clone. Mutation rate can be determined experimentally and hence provides a non-arbitrary approach to separating SSRs into different classes. The determination of mutation rates is, however, difficult and can be confounded by under- or over-estimating the number of divisions (*i.e.*, bacterial generations) relative to mutational events [5]. Nevertheless, the mutation rate of an SSR is proportional to the repeat number and influenced by the repeat unit length/sequence [15]. Hence, the repeat number can be used to separate SSRs into different ‘functional’ classes, which we have delineated as hypermutable, mutable and proto-mutable. Although, the boundaries for these classes are not easily defined and are partially arbitrary, the classifications provide a framework for distinguishing the evolved loci subject to localised hypermutation from those SSRs whose occurrence is influenced by genome composition. These classes are described below, with Table 1 providing predictions of the mutation rates and SSR tract lengths for each class.

Hypermutable SSRs consist of multiple repeat units that reproducibly generate insertions and deletions in the SSR at high rates. These hypermutable SSRs are responsible for PV. Phenotypic and molecular characterisation correlating switches in gene expression with alterations in the SSR are required to definitively ascribe a locus or specific tract length to this class of SSRs. These studies should be performed in the absence of selection, as a strong selection can amplify low frequency events, implicating short SSRs as being hypermutable and involved in PV, whereas in natural infections, such mutations would rarely mediate adaptation. Hypermutable SSRs are thought to have evolved due to frequent selection for alterations in the phenotypes controlled by mutations in the SSR, resulting in secondary selection for higher rates of mutation in the SSR (*i.e.*, longer tract lengths). These loci are likely to have evolved from other hypermutable SSRs or to be due to an increase in the tract length of a mutable SSR.

Mutable SSRs have intermediate length SSR tracts and, hence, lower mutation rates. Reversible switches in phenotypes due to these tracts may be rare, require strong selection for detection and may not always involve mutations in the SSR [16,17]. Mutable SSRs do not arise due to selection for mutability, but as a result of genome composition and the molecular forces acting on proto-mutable SSRs [18]. Thus, a higher propensity for insertions in short SSRs may result in an increase in tract length and convert a proto-mutable SSR into a mutable SSR that occasionally produces adaptive mutations.

**Table 1:** Classification of bacterial sequence repeat tracts (SSRs).

Type of SSR	Mutation Rate (Mutations/Division)	Predicted Range of Repeat Numbers (References ^1^)	
Proto-mutable	10^−9^ to 10^−7^		<6G [19]
			2-4CA [20]
			2-4TAAA
			2-4AGTC
			GACGAGAAGA
Mutable	10^−7^ to 10^−5^		6G-8G [21]
			4-10CA [20]
			3-9TAAA
			3-12AGTC [15]
			2GACGA
Hypermutable	10^−5^ to 10^−2^		>7G [21,22,23]
			>10CA[20]
			>9 TAAA[24]
			>10 AGTC [15]
			3+GACGA [25]

^1^ The indicated references contain experimental data on mutation rates for tracts of a particular type.

Proto-mutable SSRs consist of small numbers of repeat units and have mutation rates marginally higher than the genome average. These tracts are numerous in bacterial genomes and can arise by chance as a result of the nucleotide composition and codon usage of a species. These short SSRs are prone to generating mutations capable of inactivating genes, and hence, the distribution is uneven with higher numbers in the ends of genes [18]. These proto-mutable SSRs rarely produce adaptive mutations, but are the precursors for the formation of the longer tracts.

### 3.2. Detection of Multiple SSR in the Bacterial Genomes

SSR are easily detected in bacterial genomes by simple word searches and through widely-available scripts. Numerous websites offer online searches for SSRs (e.g., [26]). One of the first whole genome sequences, *Haemophilus influenzae* strain Rd, was analysed for the presence of SSRs, resulting in the identification of 12 loci subject to SSR-mediated PV; this finding represented a significant advance in our knowledge of the repertoire of phase variable genes in this organism [27]. These loci contained tetranucleotide repeat tracts consisting of five or more repeats, with the majority containing 15+ repeats. These tracts were longer than predicted to occur based solely on genome composition, suggesting their presence was due to selection for mutability.

Whole genome sequences of other important pathogens similarly identified the presence of multiple loci whose expression may be controlled by SSRs [28,29,30]. The types of SSRs vary between species, suggesting different evolutionary pathways resulting in convergence towards localised hypermutation as a mechanism of adaptation.

A major area of research is to catalogue and compare the number and distribution of SSRs and functions of SSR loci within and between species [31,32,33]. SSRs within specific loci of a species exhibit a variable presence. Thus, 55 SSR-associated loci were found in four complete *C. jejuni* genomes, but only 17 were conserved in all the genomes; and only five of these loci contained an SSR in all strains [22]. The dispersed nature of SSR-containing hypermutable genes within a species is indicative of a variable selection for these loci, due to the redundancy of their functions or a requirement for these loci for infrequently encountered selective pressures (e.g., an unusual niche or resistance to a subset of bacteriophages). Alternatively, differences in the presence or length of the SSR in a particular locus may suggest weak selection for hypermutation. More detailed analyses of genome sequences will begin to unravel the complex link between the repertoire of phase variable loci and the selective pressures driving their evolution and distribution.

## 4. Expression States, Phasotypes and the Phasevariome

### 4.1. Analysis of Expression States of Phase Variable Genes

SSRs mediate PV by altering either the transcription or the translation of a gene [4]. Alterations in repeat number of SSRs located in coding sequences result in a gain/loss of expression due to frameshifts (referred to herein as ‘translational PV’). These SSRs have to contain non-triplet repeat units, otherwise the changes have no effect on expression (although they can alter antigenicity and function, due to the insertion of extra amino acids). The majority of translational switching is between the ON and OFF, with one ON state for every two OFF states. Exceptions have been described wherein the SSR is located within the 5' end of the gene, and there are multiple initiation codons upstream of the SSR in different reading frames with differential rates of translation from these codons, producing variations in gene expression [34]. There are also cases where the SSR is at the end of the open reading frame, such that changes in the SSR alter protein length with unpredictable effects on protein function [35].

For those SSRs mediating PV through alterations in transcription, there is a different association between tract length and expression status that is not directly predictable from genomic data. The simplest type of mechanism occurs when the repeat is located between the −10 and −35 binding sites for the RNA polymerase holoenzyme. Expression levels are dependent on an optimal distance (17/18 nucleotides) between these elements, and SSRs modulate expression by altering this distance [36,37]. The result is that optimal expression is usually centred around one tract length. However, uncertainty in the actual start and end sites of the −10 and −35 motifs necessitates experimental evaluation of the expression levels of phase variants with differing tract lengths for the accurate correlation of SSR number and expression state [36]. This complicates ascertaining predictions of expression status directly from genomic sequences.

In some cases, SSRs are located upstream or downstream of the core promoter. The *nadA* gene is an example of an upstream SSR, where variation modulates interactions between repressor proteins/activator sequences and the RNA polymerase holoenzyme, thereby modulating expression [38]. A recent dissection of a downstream SSR in *Helicobacter pylori* has demonstrated a role for this SSR in modulating interactions with a small untranslated RNA (sRNA) that regulates transcript stability (S. Pernitzsch, D. Beier and Cynthia M. Sharma; Targeting of a homopolymeric G-repeat by a small RNA mediates the repression of a chemotaxis receptor in *Helicobacter pylori*. Abstract from the Campylobacter, Helicobacter and Related Organisms meeting, CHRO2013, in 2013 at Aberdeen, Scotland).

### 4.2. Phasotypes

For both translational and transcriptional PV, rules can be established linking repeat number with a particular expression status. Thus, the genotypes (repeat number) can be turned into an expression status (e.g., ON or OFF), and these states can be coded (Table 2). These expression states contain phenotypic information, but are not directly correlated with phenotype, as the expression of some of these loci can be regulated at other levels. Thus, some of the phase variable meningococcal genes are only expressed under low iron conditions [39,40]. Other phase variable genes may act to sequentially add sugar moieties, such that the phenotypic expression of the late-acting genes is dependent on the expression status of an early-acting gene. An example of this type is with the neisserial *pglA* and *pglE* genes, which act sequentially to add galactose monomers during protein glycosylation [41,42]. These coded expression states are therefore referred to as phasotypes to indicate that they are predictions of the phenotypic state of a bacterial isolate, not the actual state.

**Table 2:** Generation of a meningococcal phasotype from SSR repeat numbers.

Gene	*fetA* ^1^	*porA* ^1^	*opc* ^1^	*nadA* ^1^	*hpuA* ^2^	*nalP* ^2^
Tract Length	9C	12G	11C	9TAAA	10G	11C
Expression State^1^	2	2	1	0	2	2
Phasotype	2-2-1-0-2-2					

^1^ Genes subject to transcriptional switching such that expression states are coded into three states: high (2), intermediate (1) and low (0); ^2^ Genes subject to translational switching leading to coding into two expression states, ON (2) and OFF (0).

### 4.3. Phasotypes and the Phasevariome

**Figure 1:**
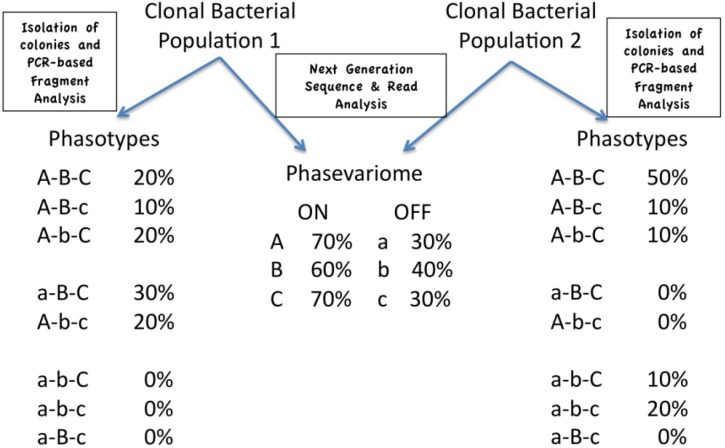
Phasevariome and phasotypes.

Analysis of the amounts of each state of a phase variable gene in a particular bacterial population is a key technique for understanding how this phenomenon contributes to adaptation and pathogenesis. In organisms with multiple phase variable genes, a population will have a particular combination of expression states for each phase variable gene—the phasevariome—whilst each cell will have a particular combination of expression states—the phasotype. As Figure 1 illustrates, the phasotypes cannot necessarily be directly predicted from the phasevariome. The analysis of the phasevariome can proceed by extracting DNA from a sample of the population and typing of the SSRs for each phase variable gene. The phasotypes, however, can only be examined by the typing of SSRs in individual cells or a surrogate—colonies grown from individual cells with the assumption that the SSRs do not change in the majority of the colony during growth. This latter approach requires plating dilutions of the population and then picking and analysing individual colonies.

Figure 1 shows the extra information contained in an analysis of phasotypes as compared to the phasevariome. The phasevariome can be obtained by next generation sequencing and describes the proportions of variants in an ON and OFF state for each gene in a population of bacterial cells. Phasotypes are obtained by the analysis of single-cell derivatives (e.g., a number of colonies grown from the population) and a PCR-based fragment analysis of each phase variable gene. Each single-cell derivative will have a particular combination of ON and OFF states for a set of phase variable genes (the phasotype). This figure depicts the analysis of a bacterial population with three phase variable genes and, hence, eight possible phasotypes. Critically, two populations with the same phasevariome can have different proportions of the eight possible phasotypes.

## 5. Analysis of SSR Diversity by Next Generation Sequencing

### 5.1. Utility of NGS for Comparison of SSR Prevalence in Bacterial Genomes

Comparisons of the prevalence of SSRs in bacterial genomes, providing an improved understanding of the evolutionary pressures acting on these sequences, has been facilitated by the NGS-driven increase in genome sequences from a wide range of species [22,33]. Similarly, strain-to-strain comparisons have also been transformed with an explosion in the numbers of genome sequences, providing the raw materials for the examination of the intra-species variability in SSR prevalence and conservation. Some studies utilising these large databases are outlined below. Nevertheless, there are problems with older and current NGS approaches, limiting the utility of some of the data for SSR tracts.

### 5.2. Methodological Impact of NGS on the Determination of Tract Length

A critical feature of an SSR tract is the repeat number, as this is a determinant of mutability [15,23]. NGS methodologies have an impact on the determination of repeat number when tracts contain large numbers of repeats or are composed of mononucleotide repeat tracts.

Long tracts of SSRs with large repeat units cannot be determined by short-read technologies, as a single read cannot span the entire repeat tract. This was a particular problem for early Illumina data, when reads were only 30 nucleotides, and so, some of the older NGS genome sequences have contig breaks in these sequences. Improvements in this technology, such as Illumina HiSeq, and the use of Roche 454 in combination with novel bioinformatics search programs have overcome this limitation, and most SSR tracts are now encompassed in NGS datasets [43].

Mononucleotide repeat tracts are, however, still a particular problem. A study of the Illumina read data for a *Drosophila* genome demonstrated that accuracy was similar to non-repetitive regions for pentanucleotide repeats, but decreased with shorter repeat units [44]. Mononucleotide tracts had the highest error rates, with internal concordance falling below the accuracy cut-off for tracts of 13 or more nucleotides. One outcome of these limitations of NGS data is that contig breaks occur frequently within phase variable genes. The analysis of meningococcal genomic data generated by Illumina indicates high error rates occurring in the polyC or polyG tracts of eight or more nucleotides, as 22 of 31 tracts were found to occur at the ends of contigs [45]. Comparisons of other recently developed platforms have also found variability in error rates and suggest that Illumina’s MiSeq may provide the greatest accuracy on repetitive tracts as compared to PacBio and Ion Torrent [46]. Thus, the determination of the actual tract length for these loci by NGS is impacted by the technology. These loci are, however, also difficult to sequence by standard Sanger-based technologies, which fail, due to a combination of the stalling of the DNA polymerase and the slippage of the DNA strands during re-initiation of DNA polymerisation, producing over-lapping sequence reads. As some NGS technologies are not reliant on a polymerase, advances in the methodology will eventually increase the accuracy across these tracts.

### 5.3. Methodological Impact of Assembly on Analysis of Multiple-Copy Loci

The potential for antigenic variation generated by phase-variable loci is enhanced by redundancies in function. The presence of two or more copies of different alleles of a phase-variable gene within a genome is common in bacterial genomes. The most extreme example is the presence of 12 alleles of the *opa* gene in *N. gonorrhoeae* genomes, which all have identical N- and C-termini, but differ in the antigenic hyper-variable regions [47]. These repetitive regions can be compromised by the NGS and/or assembly method. Distributed loci, such as the *opa* genes, can be assembled from paired-end reads, as the unique read can anchor gene reads to the correct part of a genome. *De novo* assembly may also be better than mapping to a reference genome, as mapping forces reads from different genes together. Duplicated genes in close proximity are more difficult to assemble, as the distances between paired end reads are not accurate enough to localise the exact position of a gene *versus* a unique read. These types of phase variable genes are likely to be missing from or poorly assembled in genome sequences derived using short-read technologies. The degree to which this occurs will depend on the length of the duplicated region and may need long reads of 300+ for proper assembly to occur.

## 6. Analysis of SSRs by NGS *versus* PCR-Based Approaches

### 6.1. NGS Approach

**Figure 2:**
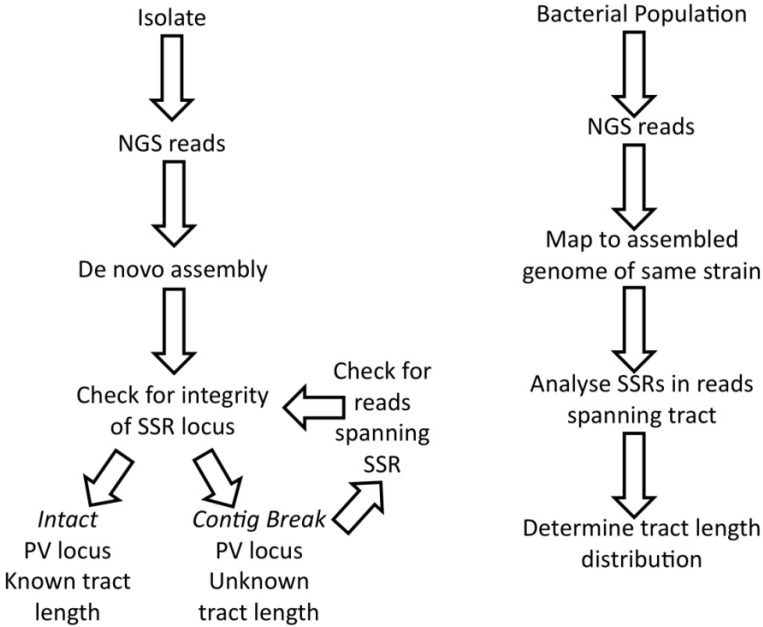
The use of next generation sequencing for the analysis of SSRs in isolates and bacterial populations. NGS, next generation sequencing. PV, phase variation.

Analysis of an SSR by NGS will provide data on the median repeat number for a population and the relative amounts of other repeat numbers when coverage is performed to a high read depth (Figure 2). Only reads containing a unique sequence from both sides of the repeat tract can be utilised for this type of analysis. The quality of the data will be influenced by the type of repeat unit, the length of the tract and the NGS methodology, as discussed above. There has, however, been no validation of such an NGS approach to compare NGS distributions of tract length to either a PCR-based approach or phenotypic characterisation (e.g., with a reporter of expression). Thus, it is unclear if systematic errors are produced by the NGS methodology. High read depths and direct analyses of samples without further growth may, however, overcome some of the caveats associated with a PCR-based approach.

Figure 2 depicts a flow chart for the analysis of SSRs in bacteria. The analysis of one isolate aims to determine the number of SSRs present in the genome and the tract lengths of these SSRs. Variation in these tracts may occur depending on how the isolate is grown prior to sequencing. Analysis of a bacterial population may be utilized following the exposure of a bacterial population to a selective pressure. The goal of this analysis is to determine the numbers of cells with particular tract lengths for a given SSR.

### 6.2. PCR-Based Approach

A PCR-based approach can be used for direct analysis of a population without growth in order to determine the commonest tract length in a bacterial population. However, slippage in the SSR during PCR amplification means that this method is inaccurate for the analysis of the relative proportions of minor tract lengths. The alternative is to analyse a subset of isolates from the population following growth in non-selective conditions. This sampling approach has obvious limitations, as a sampling error is introduced, with the magnitude of this error influenced by the number of analysed isolates. The potential for alterations in SSRs during growth is another limitation of this type of data, and the degree of selection occurring in this stage should be considered and, where possible, controlled for using comparable samples [48].

SSR analysis is performed by PCR amplification of the tract using flanking primers and then either dideoxy-sequencing of the product or sizing by a GeneScan approach. The latter method utilises one fluorescent primer and detection/sizing of fluorescent PCR products on an automated sequencing machine relative to a fluorescently-labelled DNA size standard. Repeat numbers are determined by comparison to a control sample of known repeat number (determined by sequencing) and with the same flanking sequence. This technique can detect differences of a single nucleotide, such that even mononucleotide repeat-mediated variation can be accurately quantified [22,48].

The minor advantages of this PCR-based approach are the high throughput and low cost. The major advantage is, as outlined above, the generation of information on the combined genotypes and expression states of phase variable genes, leading to the generation of phasotype data [22].

### 6.3. Use of NGS versus PCR to Analyse C. jejuni Phase Variable Genes

The genome of *C. jejuni* is AT-rich, but possesses a significant number of polyG/polyC SSR-associated genes with 12–27 in the genomes of *C. jejuni* strains, NCTC11168, RM1221, 81–176 and 81116 [22,49]. Only a subset of these genes, mainly those involved in capsule and lipooligosaccharide (LOS) biosynthesis, have been shown to be phase variable [50,51,52,53,54,55,56,57]. The lack of a mismatch repair system in *C. jejuni* translates into very high mutation rates at the polyG/C loci, ranging from 4.2 × 10^−4^ to 4.1 × 10^−3^, up to 100 times the rates of equivalent tract lengths in *N. meningitidis* [22]. The presence of multiple SSR loci and the associated high rates of switching in *C. jejuni* could potentially produce thousands of phasotypes (e.g., 2^27^ for strain NCTC11168), a huge amount of phenotypic diversity in one population. The use of both NGS and PCR-based approaches is starting to reveal the impact of this variability on the commensal and disease behaviour of this major cause of gastroenteritis.

A study by Jerome *et al.* [49] investigated alterations in the phase variable genes of *C. jejuni* strain NCTC11168 following adaptation to the infection of mice, as a model of disease. Input and output populations were subject to NGS. The prevalence of different tract lengths for each of the 28 SSRs was determined by analysis of the read data, and relative expression states were determined. An average read depth of 107 was achieved for 22 of the tracts. A subset of six loci could not be analysed by NGS, due to the high similarity between the sequences of some of these loci, preventing mapping of reads to a specific locus. These loci were analysed by PCR amplification from colonies and Sanger sequencing. Twelve genes exhibited significant differences in the expression state during adaptation to the mouse intestinal tract. Overall, this data provided the first comprehensive analysis of the changes in the *C. jejuni* phasevariome during adaptation to an animal host.

A comparable study was performed by Bayliss *et al.* [22]. This study investigated the changes in the phase variable genes of *C. jejuni* during the colonization of four-week old chickens. Six genes were analysed by a PCR-based analysis of colonies sampled from input (n = 30) and output (n = 150 or 30/bird) populations. Three were found to exhibit alterations in the expression state. Due to the colony-based approach, this study also examined the phasotypes. A total of nine and sixteen phasotypes (out of a possible 64), with non-overlapping distributions, were detected in the input and output populations, respectively. A modelling approach indicated that changes in these distributions were not due to mutational drift alone, but that selection and/or population bottlenecks must have caused the changes in the population structure. This approach has now been extended to incorporate all 27 phase variable genes of *C. jejuni* strain NCTC11168, greatly extending the potential utility of this technique [58].

The complementary nature of the NGS and PCR-based approaches suggest that both will have utility in dissecting the contributions of the phasevariome to adaptation by bacterial species with multiple genes subject to SSR-mediated PV. The additional benefit of the PCR-based derivation of phasotypes has yet to be fully explored, but has the potential to detect interacting networks of genes and provides a critical input for the modelling of the behaviour of phase variable genes.

## 7. Case Studies Illustrating Global Approaches to SSR Analysis in Clinical Isolates

A combination of genomic and experimental studies can be utilized to study the contributions of phase variable genes to bacterial virulence. The four examples described below illustrate a variety of approaches to studying how SSRs influence the asymptomatic carriage and disease attributes of bacterial pathogens.

### 7.1. Comparison of Hb-Receptor Prevalence and Expression in Meningococcal Isolate Collections

In the pathogenic *Neisseria* species, *N. gonorrhoeae* and *N. meningitidis*, scavenging of iron, an essential co-factor of many biochemical processes in living cells, is achieved by iron-regulated, surface-expressed receptors that sequester iron molecules from host glycoproteins, such as transferrin (Tf) [59,60] and haemoglobin (Hb) [61,62]. Unlike the sequences of the well-studied Tf and lactoferrin (Lf) receptors, *tbpBA* and *lbpBA*, which lack hypermutable SSRs characteristic of phase variable genes, the haemoglobin receptors, *hpuAB* and *hmbR*, contain polyG tracts that control gene expression by ON-OFF switching [63]. Genetic epidemiology studies on separate meningococcal carriage and disease isolate collections supported a direct link between the presence of the Hb receptors and the virulence of *N. meningitidis*. These studies were performed by a combination of whole genome sequencing by Illumina sequencing and PCR-based amplification/Sanger dideoxy sequencing of products. In one study by Harrison *et al.* [64], genotypic analysis of 761 meningococcal isolates (314 disease, 447 carriage) from three separate collections detected an over-representation of *hmbR* in disease isolates, especially the hyper-invasive ST-4, ST-5, ST-8, ST-11, ST-18 and ST-32 lineages, *versus* a significantly higher proportion of *hmbR*-negative isolates in the carriage group. These results implied an important role for *hmbR* and, by extension, Hb-utilization during systemic infection. Tauseef *et al.* [40] extended this understanding of the association of Hb-utilization with meningococcal disease by using a PCR-based approach to detect the presence of either only HmbR or both Hb receptors in 99% of 214 disease isolates, but an under-representation of an HpuAB only phenotype in disease as compared to carriage isolates. Finally, Harrison *et al.* [65] analysed 218 genome sequences generated by Illumina sequencing to demonstrate that HmbR is mainly restricted to pathogenic *Neisseria*, whilst HpuAB is widely distributed in both commensal and pathogenic *Neisseria* species.

**Figure 3:**
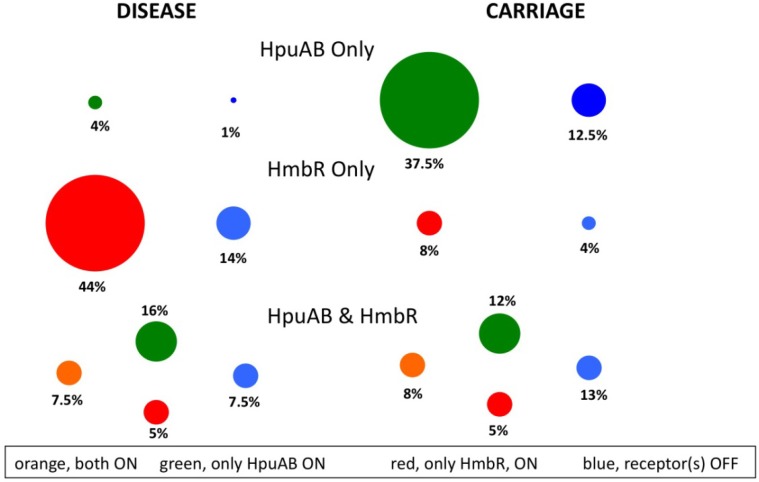
Analysis of the distributions and expression states of phase variable haemoglobin receptors in disease and carriage isolates of meningococci.

These studies revealed the prevalence of the Hb receptors (Figure 3). However, the SSRs could not be analysed, due to frequent contig breaks in the polyG tracts. Tauseef *et al.* [40] examined these tracts by a combination of dideoxy sequencing and GeneScan analysis of PCR products. These studies provided evidence of the importance of Hb receptor PV, showing that one or both receptors were in a PV-ON state (genotypic prediction of gene expression) in 91% of 90 disease isolates as compared to 71% of 103 carriage isolates. A subsequent analysis (see below) weakened this finding, as only 76% of disease isolates were found to have an Hb receptor in the ON state [48]. Differences in the strain composition of these surveys may explain these differing results.

Finally, whole genome sequencing, by a combination of whole genome shotgun sequencing and Illumina, was utilized to examine isolates from an accidental infection of a laboratory worker with an *hpuAB* and *hmbR*-positive mutator strain (*hpuAB*-OFF, *hmbR*-ON, Δ*mutS*) of *N. meningitidis* [66]. This infection led to a systemic infection of the bloodstream. Variants from this infection exhibited a loss of expression of *hmbR*, but a gain of expression of *hpuAB* via changes in the repeat tracts, thus demonstrating that SSR-associated genotypic switching of the Hb receptors occurs during infection. This switch in the homopolymeric SSRs of both genes could have been a consequence of the hypermutator phenotype, characteristic of mismatch repair-deficient (Δ*mutS*) strains [21,67], working in conjunction with the selective pressures encountered by the infecting strain during intravascular colonization of the host. The HpuAB receptor binds both Hb and Hb-haptoglobin complexes, whereas HmbR binds only Hb [68]. Perhaps the lack of free Hb molecules, due to sequestration by haptoglobin and other host Hb-binding glycoproteins, favoured the expression of *hpuAB* as disease progressed in this patient. This case provides an example of how hypermutable SSRs influence gene expression and, potentially, the virulence of *N. meningitidis*.

Two phase variable Hb-receptors, HpuAB and HmbR, are present in *Neisseria meningitidis* and are subject to translational PV, due to hypermutable polyG tracts. The majority of meningococcal strains can be separated into strains carrying both receptors or only one (*i.e.*, HpuAB only or HmbR only). This figure depicts the experimental data on the distribution and expression status of these receptors in disease (n = 80) and carriage (n = 103) isolates derived from Lucidarme *et al.* and Tauseef *et al.* [40,48], respectively. Note that the expression status was indeterminate in 1% of the disease isolates. The strain collections are not directly comparable, as serogroup B (MenB) strains predominate in the disease isolates and serogroup Y (MenY) strains in the carriage isolates, due to a paucity of analyses of MenB carriage isolates and infrequent association of MenY strains with disease.

### 7.2. Analysis of Clinical Samples from Meningococcal Patients for Hb-Receptor Expression Status

A fundamental issue in the aforementioned genetic studies on SSR-mediated PV of meningococcal OMPs is the use of isolates that have been passaged in laboratory media. This transition from an *in vivo* to an *in vitro* environment could force adaptive changes, some of which may involve PV genes. While the studies described above utilized isolates that had been minimally passaged, the likelihood of obtaining “false” *in vivo* PV states was one issue needing clarification for proper interpretation of data generated from genetic analyses. The use of clinical specimens from meningitis or septicaemia sufferers, *i.e.*, cerebrospinal fluid (CSF) or blood samples, as a substitute for laboratory-cultured isolates was pursued by Lucidarme *et al.* [48]. A nested PCR protocol was devised to amplify target PV genes from blood and CSF samples. Using this method, it was shown that minimally passaged isolates are good predictors of the *in vivo* PV status of a gene, as the data from isolates matched results from cognate clinical specimens. The genotypic predictions of *in vivo* gene expression were also largely consistent with data from phenotypic analyses. However, genetic studies often analyse a single isolate from a clinical sample and doubts have arisen as to whether data from a single isolate is representative of the entire population of meningococcal cells within a patient. In the absence of clinical specimens to confirm data generated from the analysis of a single isolate, the analysis of multiple isolates will be required in order to determine the *in vivo* ratios of PV states (e.g., relative amounts of ON and OFF states for a phase variable gene). Employment of advanced NGS methods to directly analyse clinical samples may also facilitate the generation of this type of data.

### 7.3. Phase Variable Autotransporters in Clinical Meningococcal Isolates

**Figure 4:**
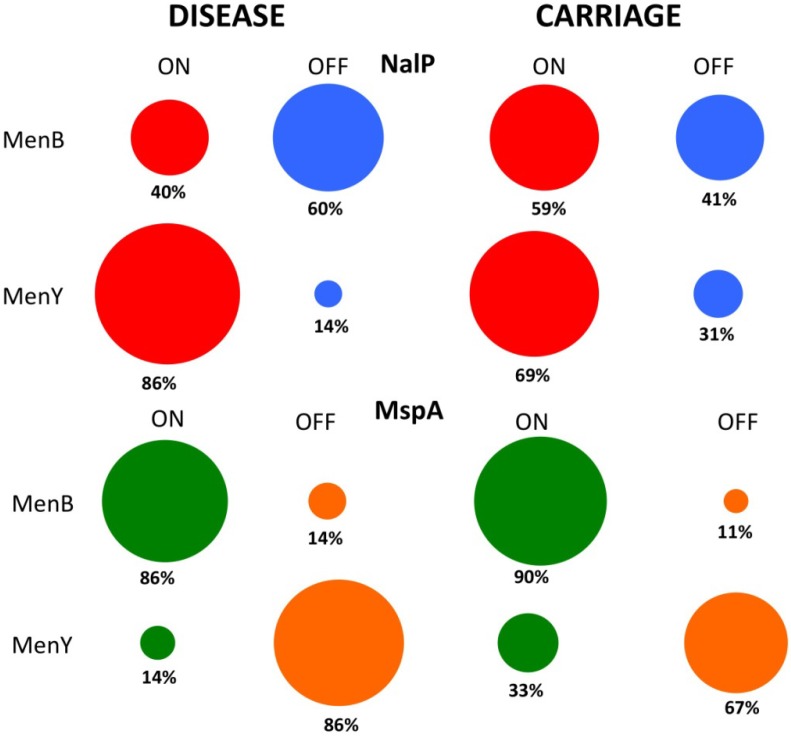
Contrasts in the expression states of two phase variable autotransporters in serogroup B and Y strains of *Neisseria meningitidis*.

Further genetic epidemiology studies on other PV genes of *N. meningitidis* have utilized genome libraries, in addition to conventional PCR and dideoxy sequencing methods, to produce interesting data that may impact on vaccine design and development. For example, a recent study by Oldfield *et al.* [69] on the autotransporters, *mspA* and *nalP*, showed high prevalences and differing expression states of either or both genes in carriage (n = 127) as compared to disease (n = 514) isolates (Figure 4). The epidemiological data on these autotransporters in disease isolates was derived by bioinformatic studies of the 500+ meningococcal genomes present in the Meningitis Research Foundation Meningococcus Genome Library. These sequences were generated using an Illumina system, and each genome is fragmented into 150–250 contigs. As expected, use of these incompletely assembled genomes translated into an inability to obtain all of the required sequences, such as for assigning all *nalP*-negative isolates to one of four *nalP* deletion classes. Crucially, SSR tract lengths were obtained from 500 *mspA* and 430 *nalP* genes from these genome sequences of invasive isolates. This high level of coverage of these phase variable genes and their associated SSRs demonstrates the potential for using these genome libraries to search for SSRs and to derive putative expression states of phase variable virulence factors.

The study by Oldfield and co-workers uncovered a potential bias for the PV-ON state of *mspA* in serogroup B strains (86%) and *nalP* in serogroup Y strains (86%), suggesting an important role for *mspA* or *nalP* during meningococcal disease, due to serogroup B and Y strains, respectively (Figure 4). While the biological significance of *mspA* expression and activity is still unclear in the meningococcus [70,71], *nalP* PV was recently found to exert a significant impact on biofilm formation and complement resistance in the meningococcus. NalP proteolytically cleaves the eDNA-binding 4CMenB vaccine target *Neisseria* heparin binding antigen (NHBA) [72], thereby reducing the eDNA-binding capacity of NalP-expressing meningococcal cells [73]. Recently, NalP was also shown to cleave C3, thereby increasing the serum resistance of meningococci [74]. Having high amounts of NalP on the meningococcal surface, as a consequence of the PV-ON state of *nalP,* may therefore have a profound effect on biofilm formation and the survival of complement-mediated killing of these serogroup Y strains, resulting in changes in their ability to persist in hosts and to cause disease. Furthermore, the expression of NalP in these serogroup Y strains and 40% of invasive MenB strains could impact on vaccine efficacy by reducing the surface expression of NHBA. However, Oldfield *et al.* maintain that the apparent lack of *nalP* expression in the majority of serogroup B strains, either by complete deletion of the gene or by PV, should not affect the use of 4CMenB for the prevention of serogroup B disease, as there is evidence that NalP deletion mutants are still susceptible to bactericidal NHBA antibodies.

The data for Figure 4 was derived from an analysis by Oldfield *et al.* [69] using a database of genomic sequences to determine expression states in disease strains (312 MenB and 73 MenY isolates for NalP; 381 MenB and 73 MenY isolates for MspA) and direct experimental analysis for carriage isolates (17 MenB and 52 MenY isolates for NalP; 19 MenB and 52 MenY isolates for MspA). Both of these autotransporters are subject to translational PV, due to a polyC tract.

### 7.4. Alterations in Outer Membrane Protein of Neisseria meningitidis and Haemophilus influenzae

Key to increased intra-host persistence of both commensal and pathogenic bacterial strains in specific niches of their hosts is the generation of a phenotypically-diverse population of cells via PV. Evidence for this assertion has come from studies of phase variable surface proteins in *N. meningitidis* and *H. influenzae* that have utilized PCR-based approaches to analyse multiple isolates from healthy or diseased individuals*.* For example, the expression of the immunogenic high-molecular-weight adhesins of *H. influenzae*, *hmw1A* and *hmw2A* is transcriptionally controlled by a heptanucleotide tract [75]. During chronic infections of the respiratory tract by *H. influenzae*, the expression levels of HMW1 and HMW2 were found to decrease as the bacteria persisted within a host. These decreases in protein expression correlated with increases in the lengths of the repeat tracts within the promoters of these genes. This decreased expression was correlated with elevated levels of anti-HMW1 and anti-HMW2 antibody titres [76]. Data from this study supports the theory that a PV-mediated reduction in gene expression allows for immune avoidance, *in vivo*. Similarly, longitudinal isolates obtained from healthy volunteers in a six-month meningococcal carriage study [77] were examined for differences in the expression of phase variable OMPs. These isolates differed in the SSR tract lengths and expression status of these OMPs with a trend of switching towards phasotypes with lower expression levels for combinations of eight phase variable surface proteins during persistent carriage [78]. The study by Alamro *et al.* analysed up to 20 isolates per time point, thereby, providing a thorough assessment of the changes in the meningococcal phasotypes and phasevariomes during carriage. The association between immune avoidance and phase variable expression state during meningococcal carriage, and indeed disease, is yet to be expressly confirmed, but the selection for lower expression states of some meningococcal OMPs is most likely due to the activity of antibodies targeting these OMPs, either individually or synergistically.

## 8. Summary

The first stage of pathogenomics has placed the emphasis on comparisons between bacterial isolates from outbreaks and epidemics, disease *versus* carriage, different isolation sites within a host and alternate types of disease presentation. These samples have begun to be exploited to determine the influence of phase variable genes on the pathogenesis and commensal behaviour of a range of bacterial species. However, the population diversity inherent in mechanisms of localized hypermutation requires a population-based approach. Future studies will place a greater emphasis on isolation and analysis of total DNA from a particular sampling point or genomic studies of multiple isolates from these samples. These studies are required to dissect the contributions of the phasevariome and phasotypes to bacterial pathogenesis. The utility of NGS approaches will assist in studies of this type of population diversity, leading to rapid and detailed analyses of the dynamic changes occurring in bacterial genomes during infections.
